# Evaluating cell viability assessment techniques: a comparative study of flow cytometry and fluorescence microscopy in response to bioactive glass exposure

**DOI:** 10.1186/s12938-025-01452-y

**Published:** 2025-10-03

**Authors:** Bolaji J. Samuel, Zhaorui Jin, Delia S. Brauer, Georg Matziolis, Victoria Horbert

**Affiliations:** 1 University Hospital Jena, Professorship of Orthopaedics at Campus Eisenberg, Friedrich Schiller University Jena, Klosterlausnitzer Strasse 81, 07607 Eisenberg, Germany; 2 Otto Schott Institute of Materials Research, Friedrich Schiller University, Lessingstraße.12 (AWZ), 07743 Jena, Germany

**Keywords:** Biomaterial, Bioactive glass, Flow cytometry, Fluorescence microscopy, Cell viability, Particle size effect

## Abstract

Reliable in vitro cytotoxicity assessment of biomaterials is essential for preclinical evaluation. Fluorescence microscopy (FM) and flow cytometry (FCM) are widely used methods, yet their comparative performance in particulate systems remains underexplored. This study compares FM and FCM in evaluating the cytotoxicity of Bioglass 45S5 (BG) on SAOS-2 osteoblast-like cells across different particle sizes and concentrations. The cells were treated with BG particles in three size ranges (< 38 µm, 63–125 µm, and 315–500 µm) at concentrations of 25, 50, and 100 mg/mL for 3 and 72 h. FM employs FDA/PI staining to distinguish viable and nonviable cells, whereas FCM utilizes multiparametric staining (Hoechst, DiIC1, Annexin V-FITC, and PI) to classify viable, apoptotic, and necrotic populations. Both techniques confirmed a clear trend: smaller particles and higher concentrations caused greater cytotoxicity. The most pronounced effect was observed for < 38 µm particles at 100 mg/mL, which reduced FM-assessed viability to 9% at 3 h and 10% at 72 h; FCM measurements under the same conditions revealed 0.2% and 0.7% viability, respectively. The controls retained > 97% viability, indicating size-based cytotoxicity. A strong correlation between FM and FCM data (*r* = 0.94, *R*^2^ = 0.8879, *p* < 0.0001) was observed. FCM further distinguished early and late apoptosis from necrosis and demonstrated superior precision, particularly under high cytotoxic stress. These findings highlight the size- and dose-dependent cytotoxic potential of BG and support the use of FCM as a robust, quantitative tool for cytocompatibility evaluation in particulate biomaterial research.

**Supplementary Information:**

The online version contains supplementary material available at 10.1186/s12938-025-01452-y.

## Introduction

Assessing the cytotoxicity of biomaterials in vitro requires reliable cell viability assays. In practice, viability is commonly determined by fluorescent live/dead stains visualized either by microscopy or by flow cytometry [1–3]. Each approach has trade-offs: fluorescence microscopy (FM) allows direct imaging of cells, whereas flow cytometry (FCM) provides high-throughput single-cell analysis. In biomaterial research, FM and FCM are both recommended techniques, but their comparative performance in particulate systems is not well established [1, 4]. Fluorescence imaging can be impeded by background signals from the material, and manual image analysis limits throughput. In contrast, flow cytometry offers rapid, quantitative viability counts but requires cells to be in suspension and access specialized instrumentation [1, 2, 5].

FM is a key imaging tool in biological research, enabling visualization of specific molecules or structures in cells and tissues. It works by exciting fluorescent dyes or proteins (fluorophores) with light, causing them to emit detectable light at a longer wavelength [6]. Conventional widefield fluorescence microscopy illuminates the entire sample, capturing emitted light through an objective lens [7]. While limited by the diffraction barrier (approximately 200 nm resolution), it remains a fundamental tool for studying protein localization, cellular dynamics, and multilabel imaging [8]. Although it is highly effective for assessing cell viability, fluorescence microscopy has several limitations [1]. These include a shallow depth of field, the risks of photobleaching and phototoxicity, interference from autofluorescence, and difficulties in accurately distinguishing between live and dead cells [9]. False positives and negatives, difficulties in quantification, resolution limitations, and the need for meticulous sample preparation contribute to the complexity of the method [10]. Manual counting or image analysis is labour intensive, which can undermine precision and throughout. The cost and expertise requirements, coupled with an inability to consistently differentiate apoptosis from necrosis, further constrain its utility [1, 11, 12].

Flow cytometry is a well-established technique in both biomedical research and clinical diagnostics. In contrast to FM, the working principle of FCM involves the analysis of cells or particles suspended in a fluid as they pass through a laser beam. The cells are labelled with fluorescent markers, and the emitted light is detected, providing information about cell characteristics [13, 14]. FCM viability assays use fluorescent probes (e.g., propidium iodide and calcein-AM) to distinguish live and dead cells, yielding precise viability percentages [15]. For example, Kummrow et al. [16], in their study on monocytes, reported that image-based tests can match flow cytometry in viability assessment and are best combined for evaluating seeding and tissue growth [16]. However, Lemos et al. [17] suggested that FCM was more precise in the determination of pancreatic islet cell viability [17]. FCM involves the detection of scattered and emitted light from particles suspended in a fluid as they pass individually through a laser beam within a flow stream [18, 19]. FCM utilizes light scattering properties, such as forward scatter (FSC) at small angles (proportional to the cell size) and side scatter (SSC) at orthogonal angles (proportional to the cell granularity), to assess differences in cell morphology. Additionally, fluorochrome-conjugated polyclonal and monoclonal antibodies, along with fluorescent dyes, have been developed and extensively employed for the analysis of highly specific biological markers [16, 20]. These markers aid in identifying and measuring the relative levels of macromolecular components on and within cells, such as DNA and various types of intracellular and cell surface antigens, thus making FCM a more robust and informative method for cellular analysis [21, 22]. FCM has emerged as an exceptionally powerful tool for investigating the distinctive features of individual cells within a heterogeneous population, eliminating the need for physical separation. This technique enables the analysis of multiple parameters related to the cell phenotype and function across various domains. Surprisingly, despite its widespread use, FCM has seen limited application in the field of biomaterial science until now [23–25].

Despite the routine use of FM for testing particulate biomaterials, its limitations in such contexts are underappreciated. Biomaterials (especially polymers and glasses) can exhibit strong autofluorescence and light scattering that “inhibit fluorescence imaging” and limit analysis of attached cells [26]. Furthermore, FMs typically sample only a few fields of view, leading to sampling bias and low throughput [17]. In contrast, flow cytometry has the potential to overcome these issues by rapidly analysing large numbers of cells and providing objective quantification [27]. In other fields (e.g., sperm and tissue graft analysis), FM has been noted as “time-consuming” and limited in terms of cell count, whereas FCM yields fast, objective results for large samples [1]. Another study named FCM time-consuming because of the long processing time compared with FM [16]. However, direct comparisons of FM and FCM for particulate biomaterial‐induced cytotoxicity are scarce. Hence, further research directly comparing FM and FCM in the context of particulate biomaterial–induced cytotoxicity is needed. Such comparative analyses are essential for establishing standardized, reliable methods for evaluating the biocompatibility of particulate biomaterials.

While numerous studies have explored bioactive glass cytocompatibility, few directly compare flow cytometry and fluorescence microscopy under controlled and identical experimental conditions. Unlike prior works that focus on biological pathways or material innovation [28], our study addresses a methodological gap by evaluating the consistency, sensitivity, and practicality of two standard viability assays in the context of particulate biomaterials.

In this study, we addressed this gap by performing a comparative evaluation of FM and FCM viability assays in an in vitro particulate system to analyse bioactive material cytotoxicity to osteoblast-like cells. For such a comparative study, Bioglass 45S5 (BG) can be used as the test material. BG is the prototypical bioactive glass (45% SiO₂, 24.5% Na₂O, 24.5% CaO, and 6% P₂O₅) that bonds to bone by forming hydroxyapatite [29–32]. It is widely used in bone repair, with over 1.5 million patient treatments reported worldwide [33], due to its osteoconductive and osteogenic behaviour [34]. Importantly, as BG dissolves, it releases Na⁺ and Ca^2^⁺ ions, which increase the local pH; this pH increase contributes to dose‐dependent cytotoxic effects by disrupting cellular homeostasis [33, 35–37]. By varying the BG particle size and concentration, one can impose a controlled gradient of cytotoxic stress on cells. This makes BG an ideal model particulate: we are therefore not characterizing its biological properties per se but rather using it to generate measurable differences in viability for FM and FCM method comparisons.

For our in vitro model, we used the human osteosarcoma cell line SAOS-2, which has a mature osteoblast-like phenotype and is widely used in bone tissue engineering studies [3]. Its robust expression of osteogenic markers and capacity to mineralize the matrix in culture make it particularly suitable for evaluating the cytotoxic effects of bioactive materials.

We hypothesize that FCM outperforms FM in terms of sensitivity, statistical resolution, and subpopulation distinction under identical experimental conditions when SAOS-2 osteoblast-like cells treated with BG are used.

We exposed the cells to BG particles of different sizes and doses, stained them for live/dead markers, and assessed their viability via both microscopy and flow cytometry under identical conditions.

Our goal was to determine how effectively each method captures the range of cytotoxic responses induced by glass. By quantifying any discrepancies between the FM and FCM results, we aim to inform best practices for viability testing of particulate biomaterials.

## Results

### pH variations

**Fig. 1:**
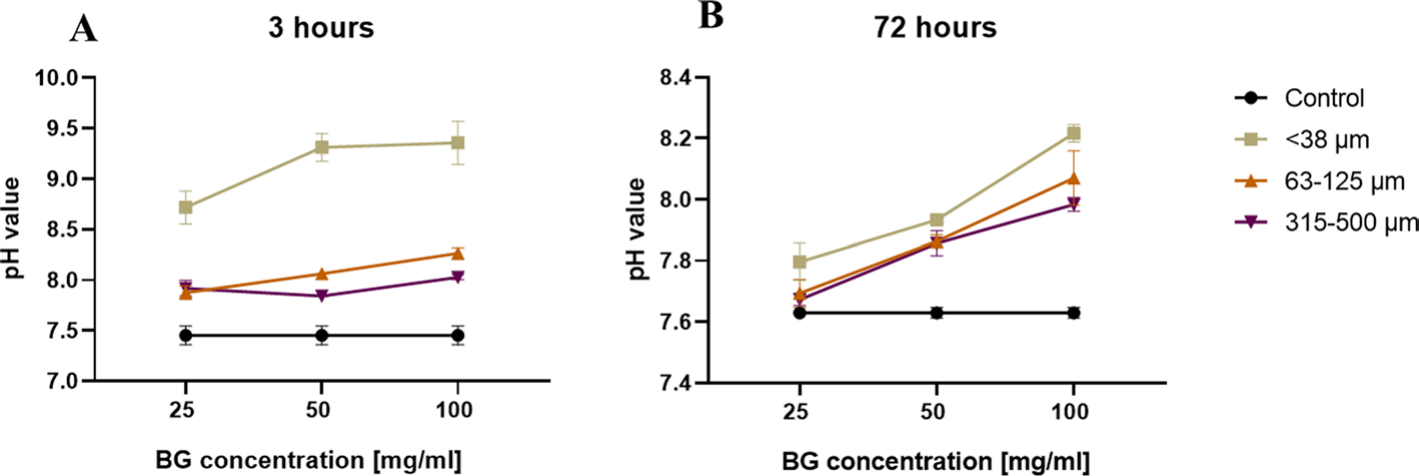
pH changes in DMEM induced by BG particles of different sizes and concentrations after **A** 3 h and **B** 72 h of incubation at 37 °C. Smaller particle sizes and higher concentrations resulted in greater alkalization, reflecting enhanced ion release. Black circles: untreated control; green squares: < 38 µm; orange triangles: 63–125 µm; purple inverted triangles: 315–500 µm. The values represent the means ± standard deviations (*n* = 3). Statistical analysis was performed via two-way analysis of variance (ANOVA) with Bonferroni correction (*p* < 0.05)

The pH of the culture medium increased with decreasing particle size and increasing Bioglass 45S5 concentration. Notably, particles < 38 µm at 100 mg/mL presented the highest pH value (9.40 ± 0.2) at the 3-h timepoint, indicating pronounced ionic exchange activity. At 3 h, all particle sizes significantly increased the pH compared with that of the untreated control (7.45 ± 0.1) across concentrations: 25 mg/mL (*p* < 0.0001, *p* = 0.0005, and *p* = 0.0001 for < 38 µm, 63–125 µm, and 315–500 µm, respectively), 50 mg/mL (*p* < 0.0001 for all sizes), and 100 mg/mL (*p* < 0.0001 for all sizes, two-way ANOVA) (Fig. 1A).

Similarly, after 72 h of incubation, a comparable pH increase was observed for 25 mg/mL (*p* = 0.0003, *p* = 0.6486, and *p* > 0.9999 for < 38 µm, 63–125 µm, and 315–500 µm, respectively), 50 mg/mL (*p* < 0.0001 for all sizes), and 100 mg/mL (*p* < 0.0001 for all sizes, two-way ANOVA) (Fig. 1B). However, the magnitude of the pH increase at 72 h was generally lower than that at 3 h, likely due to the exchange of medium after 48 h.

Overall, the pH values followed a particle size-dependent trend, decreasing with increasing particle size: < 38 µm > 63–125 µm > 315–500 µm. At 3 h, < 38 µm particles reached pH values of 8.72, 9.31, and 9.40 for 25, 50, and 100 mg/mL, respectively, whereas 63–125 µm particles presented pH values of 7.88, 8.06, and 8.27, and 315–500 µm particles presented pH values of 7.92, 7.84, and 8.03, respectively (*p* < 0.0001 for all comparisons, two-way ANOVA). Similarly, at 72 h, < 38 µm particles presented pH values of 7.80, 7.93, and 8.22, whereas 63–125 µm particles presented pH values of 7.69, 7.86, and 8.07 (*p* = 0.0106, *p* = 0.1151, and *p* = 0.0004, respectively), and 315–500 µm particles presented pH values of 7.67, 7.86, and 7.98 (*p* = 0.0022, *p* = 0.0735, and *p* < 0.0001, respectively, two-way ANOVA).

### Fluorescence microscopy outcomes

**Fig. 2:**
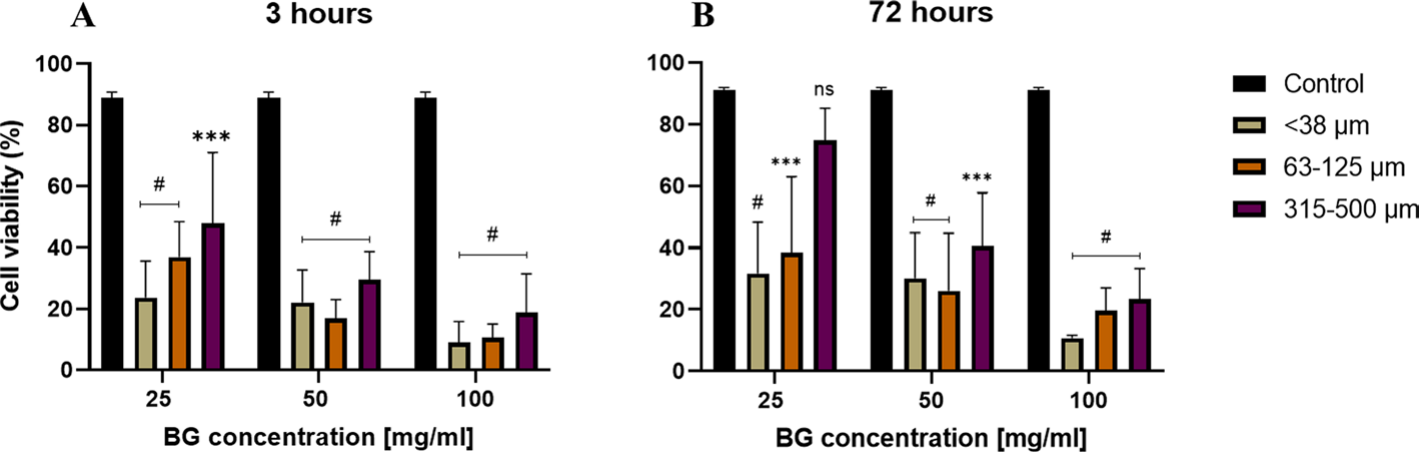
Effects of BG particle size and concentration on SAOS-2 cell viability after **A** 3 h and **B** 72 h of incubation at 37 °C, as measured via fluorescence microscopy with FDA/PI live/dead staining. Smaller particles and higher concentrations induced greater cytotoxicity, whereas larger particles exhibited lower cytotoxic effects. After 72 h, all the BG groups showed reduced cytotoxicity compared with the 3-h timepoint. The black bars represent the untreated control; the green bars represent < 38 µm particles; the orange bars represent 63–125 µm particles; and the purple bars represent 315–500 µm particles. The data are presented as the means ± standard deviations (*n* = 3). Statistical significance was determined via two-way ANOVA with Bonferroni post hoc correction (*p* < 0.05); # = *p* < 0.0001, *** = *p* = 0.0005, *ns* not significant (*p* > 0.9999)

As an initial assessment of cytotoxicity and cell viability in the osteoblast-like SAOS-2 cell line, FM was performed on all samples treated with BG particles of varying sizes (< 38 µm, 63–125 µm, and 315–500 µm) and concentrations (25, 50, 100 mg/mL), alongside untreated controls and other relevant controls. Following exposure, FM analysis revealed a significant, size- and concentration-dependent decrease in SAOS-2 cell viability at both 3 and 72 h (Fig. 2A, 2B).

At 3 h, the FM results indicated a notable reduction in viability for all particle sizes and concentrations compared with the untreated control (88.8% ± 2.1%, *p* < 0.0001). The viability values of the cells exposed to < 38 µm particles were 23.7% ± 11.0%, 22.1% ± 10.6%, and 9.8% ± 6.8% at 25, 50, and 100 mg/mL, respectively (*p* < 0.0001 for all concentrations, two-way ANOVA). Similarly, viability following exposure to 63–125 µm particles was 37.0% ± 11.9%, 17.0% ± 6.1%, and 10.5% ± 4.5% for 25, 50, and 100 mg/mL, respectively (*p* < 0.0001 for all concentrations, two-way ANOVA). In contrast, the viability of the cells treated with 315–500 µm particles was 50.2% ± 18.0%, 29.5% ± 9.2%, and 18.7% ± 5.8% at the corresponding concentrations (*p* = 0.0007, *p* < 0.0001, and *p* < 0.0001, respectively, two-way ANOVA). Although some variability was observed among replicates, particularly for smaller particle sizes, the overall trend consistently demonstrated that smaller particles and higher concentrations demonstrated the highest cytotoxicity.

At the 72-h timepoint (Fig. 2B), the cell viability improved across all the groups relative to the 3-h results. The viability of the cells treated with < 38 µm particles at 100 mg/mL was 10.7% ± 0.8%, whereas that of the untreated control cells was 91.1% ± 0.8% (*p* < 0.0001). Similarly, the viability of cells treated with 315–500 µm particles at 100 mg/mL increased to 25.0% ± 9.8% *(p* < 0.0001).

**Fig. 3:**
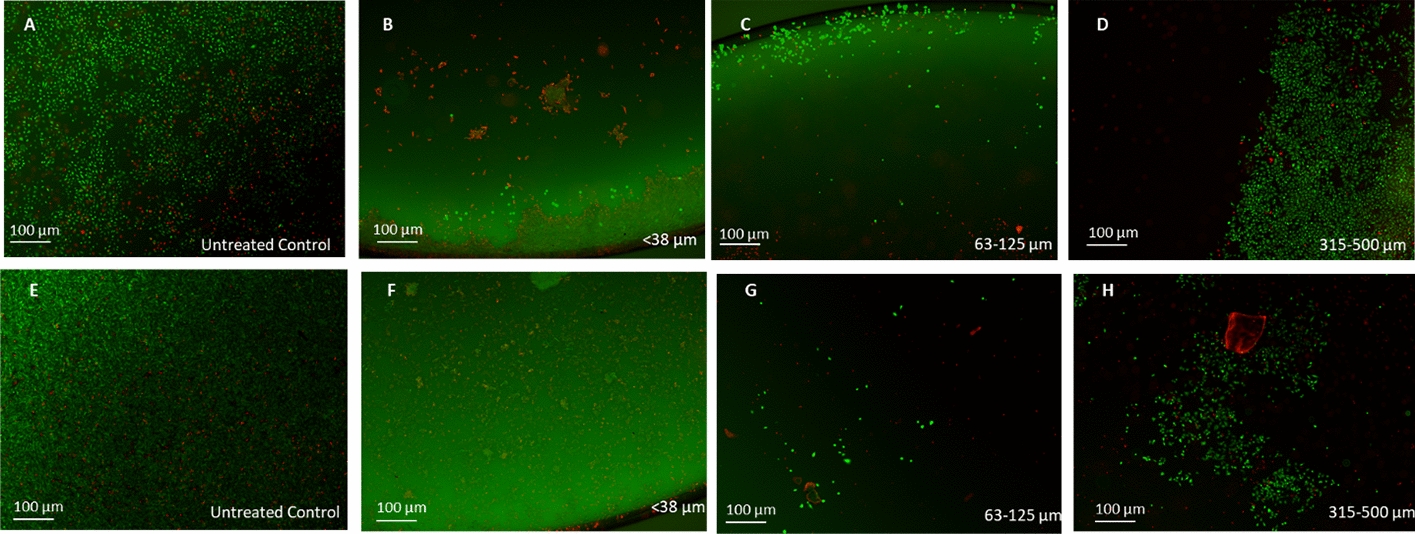
Impact of the BG particle size on SAOS-2 cell viability. Representative fluorescence microscopy images of cells after 3 h (**A**–**D**) and 72 h (**E**–**H**) of direct contact with 45S5 particles stained with fluorescein diacetate (FDA, green; viable cells) and propidium iodide (PI, red; nonviable cells) via a live/dead assay. Conditions: **A** untreated control, **B** < 38 µm particles, **C** 63–125 µm particles, and **D** 315–500 µm particles

Representative fluorescence microscopy images (Fig. 3) confirmed the decrease in SAOS-2 cell viability with decreasing particle size and increasing BG concentration after 3 h and 72 h of incubation.

### Flow cytometry outcomes

**Fig. 4:**
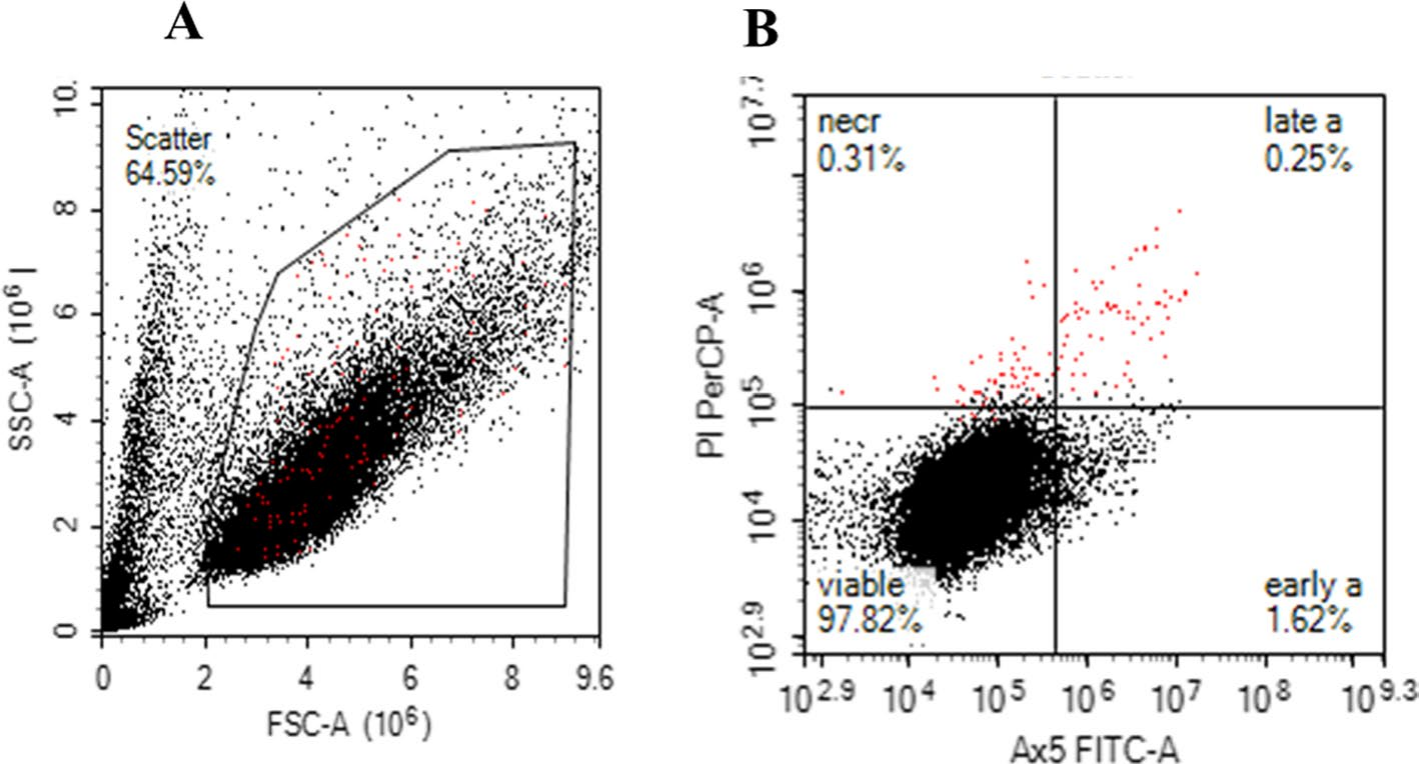
Representative flow cytometry scatter plots illustrating the gating strategy used to assess intact SAOS-2 cell populations after 3 h of incubation under control conditions (without BG). **A** Forward scatter (FSC) versus side scatter (SSC) plot identifying intact single cells. Red events indicate propidium iodide (PI)-positive cells, whereas black events represent Hoechst-positive, DilC1-positive, and Annexin V-FITC–positive (Ax5 +) cells. **B** Annexin V-FITC versus PI scatter plot displaying viable, early apoptotic (early a), late apoptotic (late a), and necrotic (necr) populations. Cell phases are expressed as percentages of total gated events. Early apoptotic cells are positive for Annexin V-FITC only, whereas late apoptotic and necrotic cells are double-positive for both Annexin V-FITC and PI

**Fig. 5:**
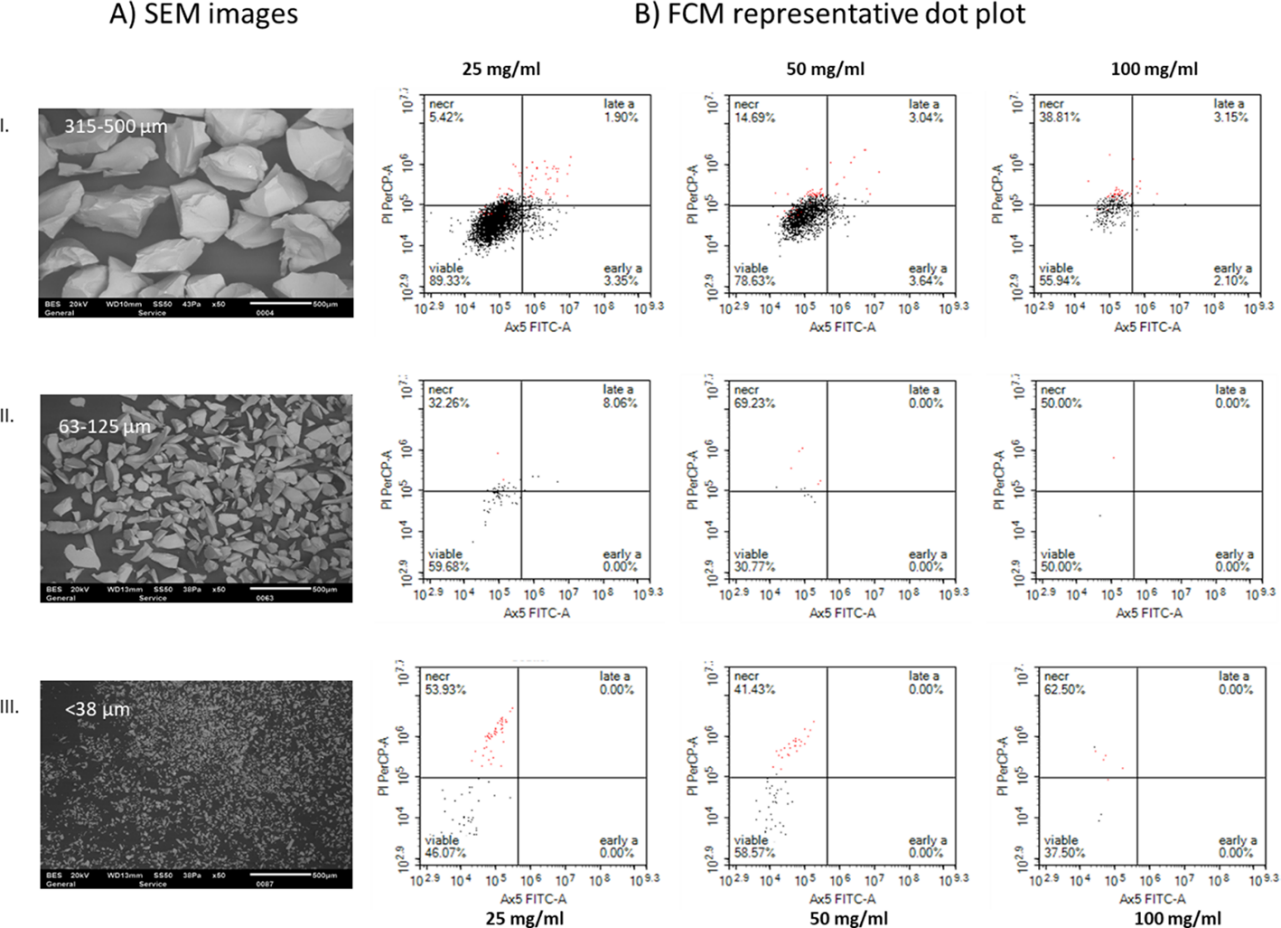
Impact of the BG particle size on SAOS-2 cell viability. **A** SEM images showing BG particle sizes: I. 315–500 µm, II. 63–125 µm, III. < 38 µm. **B** Representative FCM dot plots of cells after 3 h of direct contact with 45S5 particles (I, II, and III) at different concentrations (25, 50, and 100 mg/mL, respectively). The density of captured events decreased with increasing BG concentration and decreasing particle size, with smaller BG particles exhibiting greater cytotoxicity. Flow cytometry analysis was performed on the basis of the previously described gating strategy (Fig. 4), excluding debris. Annexin V-FITC versus PI scatter plots showing viable, early apoptotic (early a), late apoptotic (late a), and necrotic (necr) cells

Owing to autofluorescence interference caused by BG particles, as observed via fluorescence microscopy, flow cytometry (FCM) was employed to provide a more quantitative analysis of SAOS-2 cell viability. Gating was applied to exclude debris and identify intact cell populations via forward scatter (FSC) versus side scatter (SSC) parameters (Fig. 4). The staining profiles distinguished viable (Hoechst⁺/DilC1⁺), early apoptotic (Hoechst⁺/DilC1⁺/Annexin V-FITC⁺/PI⁻), late apoptotic (Hoechst⁺/Annexin V-FITC⁺/PI⁺), and necrotic (Annexin V-FITC⁺/PI⁺) populations. From the gated events, FCM enabled percentage-based discrimination of cell death phases across different particle sizes and concentrations at both 3 and 72 h (Fig. 5).

At the 3-h time point, the untreated control group contained 97.5% ± 0.1% viable cells, 1.5% ± 0.5% early apoptotic cells, 0.3% ± 0.1% late apoptotic cells, and 0.6% ± 0.3% necrotic cells (Table 1). In comparison, exposure to < 38 µm particles at 25 mg/mL resulted in 78.2% ± 10.9% viable cells, 1.0% ± 1.1% early apoptotic cells, 0.4% ± 0.6% late apoptotic cells, and 18.4% ± 9.8% necrotic cells. Similarly, cells exposed to 63–125 µm particles at 25 mg/mL presented 77.6% ± 15.9% viability, 7.7% ± 9.7% early apoptotic, 5.9% ± 8.2% late apoptotic, and 8.8% ± 2.2% necrotic cells. For the 315–500 µm group, the viability was 70.4% ± 17.9%, with 15.8% ± 14.4% necrotic cells.

**Table 1:** SAOS-2 cell death phases assessed by flow cytometry (FCM) after exposure to BG particles of varying sizes (< 38 µm, 63–125 µm, and 315–500 µm) and concentrations (25, 50, and 100 mg/mL) for 3 h and 72 h of incubation

Observed values by FCM						
Timepoint	Particle size (µm)	Conc. (mg/mL)	Viable (%) Mean ± SD	Early apoptotic (%) Mean ± SD	Late apoptotic (%) Mean ± SD	Necrotic (%) Mean ± SD
3 hours	Control		97.5 ± 0.1	1.5 ± 0.5	0.3 ± 0.1	0.6 ± 0.3
	<38	25	78.2 ± 10.9	1 ± 1	0.4 ± 0.6	18.4 ± 9.8
		50	37.7 ± 23	0 ± 0	0 ± 0	62.3 ± 28.2
		100	31.1 ± 21.9	0.3 ± 0.6	0.7 ± 1.1	67.9 ± 25.5
	63–125	25	77.6 ± 15.7	7.7 ± 9.7	5.9 ± 8.2	8.8 ± 2.2
		50	82.3 ± 6.2	6.5 ± 4	0 ± 0	11.2 ± 11
		100	81.6 ± 13.8	4.3 ± 5.3	0 ± 0	14.1 ± 15.5
	315–500	25	70.4 ± 17.9	7 ± 5	6.8 ± 5.3	15.8 ± 14.4
		50	74.6 ± 15.8	4.9 ± 3.2	7.4 ± 6.5	13.1 ± 9.7
		100	69.6 ± 11.7	8.7 ± 5.7	6.9 ± 3.8	14.8 ± 9
72 hours	Control		97.4 ± 0.5	2 ± 0.4	0.3 ± 0.1	0.3 ± 0.1
	<38	25	50.9 ± 8.9	0 ± 0	0 ± 0	49.1 ± 8.9
		50	47 ± 12.7	0 ± 0	0 ± 0	53 ± 12.7
		100	56.9 ± 19.2	0 ± 0	0.9 ± 1.5	42.2 ± 19.3
	63–125	25	60 ± 10.1	0.9 ± 1.6	9.6 ± 6.4	29.5 ± 5.7
		50	62.1 ± 27.3	4.6 ± 4.9	0 ± 0	33.2 ± 31.7
		100	50 ± 50	0 ± 0	0 ± 0	16.7 ± 28.9
	315–500	25	89.9 ± 4.4	3.8 ± 1.4	1.6 ± 0.7	4.7 ± 2.6
		50	82 ± 6.5	4.5 ± 1.5	2.7 ± 1.5	10.8 ± 4.6
		100	66.5 ± 16.3	2.8 ± 1.4	2.5 ± 2.3	28.2 ± 13.6

The cell populations were categorized as viable (Hoechst^+^/DilC1.^+^), early apoptotic (Annexin V-FITC⁺/PI⁻), late apoptotic (Annexin V-FITC⁺/PI⁺), or necrotic (Annexin V-FITC⁺/PI⁺) on the basis of scatter plots. Data are expressed as the percentage mean ± standard deviation of total gated events (*n* = 3)

Although these percentages reflect cell death phase distributions among gated intact cells, the pronounced cytotoxicity of BG, especially at smaller sizes and higher concentrations, resulted in significantly fewer intact cells being captured. This introduced variability and limited direct comparisons with the untreated control. To account for this, all datasets were normalized to 5,000 total events.

**Fig. 6:**
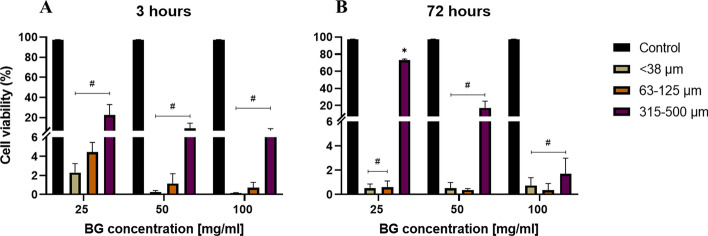
Quantitative analysis of SAOS-2 cell viability after **A** 3 h and **B** 72 h of exposure to BG particles of different sizes and concentrations, as measured via a NovoCyte flow cytometer. Cell viability was determined from gated scatter populations and normalized to 5000 events. The data are presented as the means ± standard deviations (*n* = 3). Statistical significance was determined via two-way ANOVA with Bonferroni post hoc correction (p < 0.05); #*p* < 0.0001, *p < 0.01

Further FCM analysis of the normalized data confirmed a clear size- and concentration-dependent decrease in SAOS-2 cell viability (Fig. 6). The untreated control maintained high viability at both timepoints, with 97.7% ± 0.1% and 97.4% ± 0.5% viable cells at 3 h and 72 h, respectively. In contrast, exposure to 25 mg/mL BG particles significantly reduced viability at 3 h: < 38 µm particles resulted in 2.3% ± 0.9% viability, 63–125 µm particles resulted in 4.8% ± 4.3%, and 315–500 µm particles resulted in 22.6% ± 10.2% viability (*p* < 0.0001 for all, two-way ANOVA).

At 72 h (Fig. 6B), viability remained low for the smaller particles: < 38 µm and 63–125 µm particles resulted in 0.5% ± 0.4% and 0.6% ± 0.5% viability, respectively (*p* < 0.0001 for both two-way ANOVA), whereas cells treated with 315–500 µm particles showed significantly greater viability (73.1% ± 1.1%, *p* = 0.0106, two-way ANOVA). Importantly, cell viability decreased progressively with increasing BG concentration (25 mg/mL > 50 mg/mL > 100 mg/mL), with 100 mg/mL resulting in the lowest viability across all sizes and timepoints (*p* = < 0.0001 for all particle sizes, two-way ANOVA).

### Comparative analysis

**Fig. 7:**
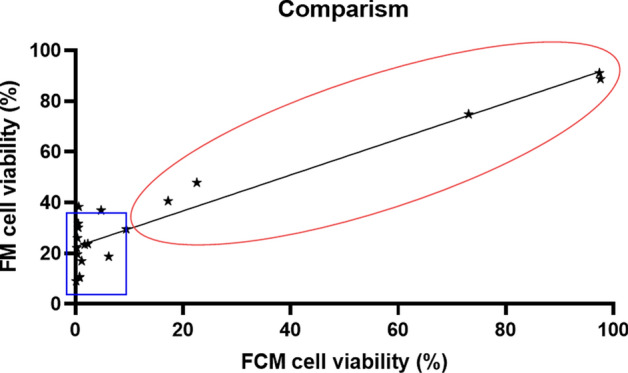
Comparison of SAOS-2 cell viability results obtained by fluorescence microscopy and flow cytometry. A strong positive correlation is observed between the two methods (*r* = 0.94), with a coefficient of determination *R*^2^ = 0.8879 and a linear regression equation: *y* = 0.7089x + 22.58. The red circles indicate regions of high measurement accuracy and reliability, whereas the blue squares highlight regions with reduced accuracy and precision

A comparison of cell viability measurements obtained by fluorescence microscopy (FM) and flow cytometry (FCM) revealed a strong positive correlation (*r* = 0.94, *R*^2^ = 0.8879, *p* < 0.0001, two-way ANOVA) (Fig. 7). Across all the BG-treated groups (three particle sizes and concentrations at both 3 and 72 h), moderate variability was observed, particularly in the low-viability samples. The coefficients of variation (CVs) remained below 10% in most cases, indicating acceptable precision (Table 2). Notably, the FCM method demonstrated reliable measurements down to approximately 20% viability, whereas FM remained consistent down to approximately 40%. Below these thresholds, both techniques showed reduced accuracy and precision.

**Table 2:** Comparison of SAOS-2 cell viability percentages under various treatment conditions, as assessed by fluorescence microscopy (FM) and flow cytometry (FCM)

Observed values								
	FCM				FM			
	3 hours		72 hours		3 hours		72 hours	
Conditions	Mean±SD (%)	CV (%)	Mean±SD (%)	CV (%)	Mean ± SD (%)	CV (%)	Mean ± SD (%)	CV (%)
Control	97.6 ± 0.11	0.1	97.4 ± 0.5	0.5	88.8 ± 2.1	2.3	91.1 ± 0.8	0.9
<38 µm [25 mg/ml]	2.3 ± 0.9	40.8	0.5 ± 0.4	69.7	23.7 ± 11.9	50.2	31.7 ± 16.4	51.8
<38 µm [50 mg/ml]	0.2 ± 0.7	68	0.5 ± 0.5	88.7	22.1 ± 10.6	47.9	30.2 ± 14.7	48.7
<38 µm [100 mg/ml]	0.2 ± 0	27.2	0.7 ± 0.6	85.4	9.0 ± 6.8	75.4	10.7 ± 0.9	8.3
63–125 µm [25 mg/ml]	4.8 ± 4.2	88.7	0.6 ± 0.5	81.9	37.0 ± 11.4	30.8	38.4 ± 24.7	64.4
63–125 µm [50 mg/ml]	1.2 ± 1.0	87.5	0.4 ± 0.1	29.3	17.0 ± 6.1	36	26 ± 18.6	71.6
63–125 µm [100 mg/ml]	0.7 ± 0.6	77.2	0.4 ± 0.5	149.8	10.5 ± 45	43.2	19.7 ± 7.4	37.7
315–500 µm [25 mg/ml]	22.6 ± 10.3	45.4	73.1 ± 1.1	1.5	47.9 ± 23	48.4	74.9 ± 10.3	13.8
315–500 µm [50 mg/ml]	9.4 ± 5.0	52.6	17.2 ± 7.8	45.3	29.5 ± 9.2	31	40.6 ± 17.2	42.5
315–500 µm [100 mg/ml]	6.2 ± 2.8	45.1	1.7 ± 1.3	75.8	18.7 ± 12.7	68.2	23.5 ± 9.8	41.8

The data are presented as the means ± standard deviations (*n* = 3). The coefficient of variation (CV) is also provided to assess measurement precision

In the untreated control groups, the CV values for FCM were exceptionally low (0.1% at 3 h and 0.5% at 72 h), whereas FM showed slightly greater variability (2.3% at 3 h and 0.9% at 72 h).

## Discussion

In this study, we compared FM and FCM for quantifying SAOS-2 cell viability following exposure to Bioglass 45S5, addressing the notable gap in direct comparative analyses of these methods for particulate biomaterial-induced cytotoxicity. Leveraging the established reactivity of BG, we created defined conditions to evaluate size- and dose-dependent cytotoxic responses at both the 3 h and 72 h time points. Our findings confirmed that cytotoxicity increased with decreasing particle size and increasing concentration, with < 38 µm particles at 100 mg/mL causing the most pronounced reduction in viability (< 5%) at both the 3 h and 72 h time points (Table 1). A strong correlation was observed between the FCM and FM viability measurements (*r* = 0.94, *R*^2^ = 0.8879; Fig. 7), indicating high consistency between the two methods. Both methods demonstrated high precision in the untreated control group (CV = 0.5% and 0.9%, respectively), and the precision varied considerably across all BG-treated samples, with coefficients of variation exceeding 10% for both techniques. Although FM offers rapid visualization and morphological assessment via FDA-PI staining to differentiate viable (green) and nonviable (red) cells, its quantitative accuracy is compromised by autofluorescence from BG particles and the subjectivity of manual scoring, even when assisted by Keyence BZ-X Analyser software [38, 39]. The superior resolution of FCM over FM in viability profiling was further evidenced by its capacity to differentiate viable, early apoptotic, late apoptotic, and necrotic populations, even under conditions of pronounced cytotoxic stress [35].

The observed cytotoxicity trend is consistent with previous reports [40, 41], indicating that smaller BG particles exhibit greater reactivity due to their greater surface area-to-volume ratio, which likely accelerates ion release and alters the local environment [35, 42, 43]. Sepulveda et al. [42] reported that melt-derived 45S5 BG (38–45 µm) exhibits slow dissolution kinetics at a specific surface area (0.5 m^2^/g), limiting ion exchange primarily to the outer surface. Nevertheless, it induces a rapid increase in pH (> 9) through the release of Na⁺ and Ca^2^⁺ ions, which significantly influence both its bioactivity and cytocompatibility [42]. These ions, particularly Na^+^, Ca^2+^, and SiO_4_-, can increase the pH and disrupt cellular homeostasis, promoting necrosis, especially in two-dimensional in vitro cultures where the BG particles are in direct contact with the cells [3, 31, 44]. In our system, this shift likely contributes to acute cytotoxicity at 100 mg/mL, especially at 3 h where peak pH was observed. The FCM profiles of these groups confirm this by showing increased necrotic gates. These findings support the role of surface area-driven ion dynamics as a key cytotoxic mechanism. This inclusion of two incubation time points (3 and 72 h) further emphasized the persistence and progression of the cytotoxic effects. Notably, at 3 h, exposure to < 38 µm particles reduced viability to 2.3% ± 0.9%, which decreased further to 0.5% ± 0.4% at 72 h. In contrast, cells exposed to 315–500 µm particles maintained substantially greater viability (22.6% ± 10.2% at 3 h and 73.1% ± 1.1% at 72 h). Although increased toxicity with smaller, more concentrated particles is expected due to higher surface area and ion release, our focus was on comparing how FM and FCM reflect this trend under identical conditions. Interestingly, FCM captured necrotic profiles more distinctly, suggesting it may better resolve dose-dependent effects in particulate systems than FM, which is more susceptible to overlapping signal interference. Prior studies have also associated small-particle BG exposure with increased oxidative stress and mitochondrial damage, which aligns with our finding of substantial viability loss and necrotic cell death [40, 45]. Furthermore, our FCM-based discrimination of death phases builds on previous literature that has emphasized the importance of multiparametric approaches when assessing apoptosis and necrosis in vitro [46–49]. Although our findings align with those of Lemos et al. [17], their study on pancreatic islet cells employed only two dyes, 7-AAD and Annexin V-APC, to identify necrotic and apoptotic cells, respectively, while unstained cells were assumed to be viable [17]. In contrast, building on their work, our study employed a four-dye staining strategy to enable a more precise classification of cell states: Hoechst 33342 was used to label all nucleated cells, DilC1 was used for identifying viable and early apoptotic cells on the basis of mitochondrial membrane potential, Annexin V-FITC was used for phosphatidylserine externalization (a marker of early and late apoptosis), and PI was used to detect loss of membrane integrity indicative of necrosis. This multiparametric staining strategy enhances the resolution and accuracy of cell death phase discrimination in flow cytometric analysis and supports a more detailed cytocompatibility evaluation of particulate biomaterials [45, 49].

In interpreting our results, it was important to consider the strengths and limitations of the analytical techniques employed. FCM enables high‐throughput, multiparametric profiling of SAOS-2 cells, distinguishing live (Hoechst⁺/DiIC1⁺), early apoptotic (Annexin V-FITC⁺/PI⁻), and necrotic (PI⁺) populations, using stringent gating to exclude debris. However, the cell harvesting and staining procedures associated with this technique can introduce mechanical stress and occasional spectral overlap, which may compromise the absolute quantification of cell death phases. Therefore, we recommend that FCM remain the primary tool for quantitative cell fate analysis in biomaterial testing, with complementary assays such as caspase activity measurements or live-cell imaging used to validate mechanistic interpretations. In contrast, FM offered rapid live/dead discrimination with valuable spatial and morphological context but remained more subjective and was susceptible to autofluorescence artefacts.

However, there are important limitations. BG particles, while serving as a well‐characterized model of cytotoxic biomaterials, represent artificial surfaces whose in vitro behavior may differ from that of complex in vivo environments [40, 50]. FM’s utility was constrained by subjective interpretation and autofluorescence artifacts from BG particles, particularly when cell viability fell below 40%. Although FCM is multiparametric and depends on the ability to harvest sufficient intact cells, severe cytotoxicity reduces event counts and introduces variability. Moreover, mechanical stress during cell detachment and potential spectral overlap in fluorescence channels may affect the absolute quantification of death phases.

We acknowledge that this investigation employed only one osteoblast‐like cell line in two‐dimensional culture, which may not fully recapitulate the behaviour of primary cells or three‐dimensional tissue contexts. Nonetheless, SAOS-2 cells have been widely validated for their ability to evaluate osteogenic responses and cytotoxicity to bone graft materials and bioactive glasses [51]. The stable expression of osteoblastic markers, mineralization capacity, and reproducibility of these materials under stress make them well suited for comparative studies. Their scalability also facilitates systematic testing across variables such as dose, timepoint, and material type. Moreover, two-dimensional cultures using osteoblast-like cell lines have shown cytotoxic responses that are consistent with those of primary cells and three-dimensional models under controlled conditions [3, 52]. We used the SAOS-2 cell line for its osteoblastic phenotype, reproducibility, and widespread use in biomaterials testing. However, as a cancer-derived line, it may differ from primary osteoblasts or mesenchymal stem cells in apoptotic sensitivity and stress signalling. While this may affect absolute cytotoxicity levels, it does not compromise the comparative value of our assay-focused analysis. Future studies should validate findings using primary cell systems.

Smaller particles and higher concentrations led to marked reductions in SAOS-2 viability, primarily via necrosis, and this effect was amplified over time. FCM has emerged as a superior method for the quantitative assessment of cell viability and death phases, whereas FM serves as a complementary, although slightly less precise, tool [26]. These results highlight the necessity of optimizing BG formulations for safe and effective application in bone tissue engineering and support the integration of multiparametric cytotoxicity assays in the evaluation of bioactive materials [31].

The choice of analytical method significantly affects the interpretation of cytocompatibility. FCM offers high resolution and quantitative reliability, making it ideal for mechanistic studies and preclinical validation, provided that there are enough intact, viable single cells and carefully selected fluorochromes. However, the requirement of FCM for large populations of viable single-cell suspensions limits its use with rare or limited samples [27]. In contrast, FM is valuable for rapid morphological assessment and initial viability screening, especially when the sample size is small or when the spatial context is important [5]. FM enables direct visual observation and can be further strengthened when combined with other orthogonal methods, supporting a more robust and comprehensive evaluation of cytocompatibility [53]. The enhanced cytotoxicity observed with high concentrations of < 38 µm particles may be attributed to several complementary mechanisms. First, the large surface area of these fine particles promotes rapid ion release and localized pH shifts, which are known to influence cell viability. Second, mechanical entrapment and physical crowding of cells by dense particle aggregates can impede cell spreading, attachment, and nutrient exchange. Sedimentation effects may further exacerbate these interactions by creating localized microenvironments where cells are surrounded by high particle densities. These factors likely act synergistically, leading to the pronounced reduction in viability observed in the < 38 µm, 100 mg/mL group. Such effects should be considered when interpreting in vitro cytocompatibility assays involving particulate biomaterials. However, it is important to recognize that these phenomena may be less pronounced in vivo, where dynamic fluid flow, immune clearance, and tissue remodelling processes can mitigate particle accumulation and sedimentation effects. We recognize that our FCM protocol incorporated multiple viability and apoptosis markers, whereas FM relied solely on FDA/PI staining; this discrepancy, while reflective of standard laboratory workflows, may have amplified FCM’s apparent advantage in subpopulation resolution. Furthermore, although rigorous methodological controls were applied, elevated particle loads can attenuate fluorescence signals in both FM and FCM, potentially affecting quantitative accuracy. To address these limitations, future work should incorporate additional FM-compatible probes (e.g., MitoTracker, DAPI) and deploy complementary approaches such as imaging cytometry or SEM-based correlative analyses to mitigate signal interference and enhance validation of cell–material interactions.

These findings underscore the importance of the FCM and FM techniques in elucidating the cytotoxic effects of BG at various particle sizes and concentrations on SAOS-2 cell viability. Each method has distinct strengths and limitations, and their complementary use can provide a more complete understanding of biomaterial and cytocompatibility studies.

## Conclusion

This study highlights the methodological advantages of flow cytometry over fluorescence microscopy in assessing the cytotoxicity induced by bioactive glass particles. A major strength of this study is its dual‐method design, which enables direct comparison of FM and FCM under identical experimental conditions. These findings suggest that both FM and FCM provide high precision in conditions with moderate to high cell viability and can serve as complementary methods for robust and accurate cytocompatibility assessments in biomaterials research. While both methods detect viability changes, FCM delivers higher resolution, better reproducibility, and detailed subpopulation profiling. FM provides rapid visualization and morphological context via FDA/PI staining but is limited in resolution and accuracy, particularly under high cytotoxic stress, due to background autofluorescence from BG particles and variability in manual image interpretation. For accurate evaluation of biomaterials, especially those producing autofluorescence, FCM should be prioritized, with many controls included, and other technique quantification techniques should be combined to validate the results.

## Materials and methods

### Glass melting and particle size preparation

BG 45S5 was synthesized via melt-quenching following established protocols [54]. The glass was milled and sieved into three size fractions: < 38 µm, 63–125 µm, and 315–500 µm. The particle size distributions were confirmed via laser diffraction analysis and scanning electron microscopy (SEM) [55].

### Cell culture and treatment

Human osteoblast-like SAOS-2 cells (osteosarcoma cell line, DSMZ—German Collection of Microorganisms, Germany) were cultured in McCoy’s 5A medium (PAN-Biotech, Germany) supplemented with 15 vol.% fetal bovine serum (FBS, Gibco, UK), 2 vol.% penicillin‒streptomycin (Pen/Strep, [10,000 U/mL and 10 mg/mL]; PAN-Biotech, Germany), and 1 vol.% gentamicin ([10 mg/mL]; Gibco, UK) at 37 °C and 5% CO_2_. The cells were grown for 48 h to confluence in 75 cm^2^ culture flasks (CELLSTAR, Germany), washed with phosphate-buffered saline (PBS) and harvested via trypsin (Gibco, UK). Initial cytotoxicity of the glasses was carried out by culturing BG particles in direct contact with SAOS-2 cells seeded in 24-well plates (100,000 cells/ml), incubated for 72 h, and treated with BG particles suspended in modified Dulbecco’s modified Eagle’s medium (DMEM; PAN-Biotech, Germany): 10 vol.% FBS, 2 vol.% Pen/Strep, 1 vol.% HEPES (10 mM; Carl Roth, Germany), 0.2 vol.% 2-phospho-L-ascorbic acid trisodium salt (0.2 mM; Sigma‒Aldrich, Germany), and 0.5 vol.% β-glycerophosphate disodium salt hydrate (10 mM; Sigma‒Aldrich, USA). Each of the three BG particle sizes (< 38 µm, 63–125 µm, and 315–500 µm) was tested at concentrations of 25, 50, and 100 mg/mL for 3 and 72 h of exposure [56, 57].

Particle sizes (< 38 µm, 63–125 µm and 315–500 µm) and concentrations (25–100 mg/mL) were selected based on prior reports evaluating cytotoxic thresholds of bioactive glass in direct-contact models [4, 58, 59]. These ranges reflect clinically relevant surface area variations and simulate dose-dependent effects. Timepoints at 3 h (early ionic exchange phase) and 72 h were chosen to represent acute and delayed cytotoxic phases, as recommended in ISO 10993-5 and related studies [28, 60].

### pH measurement protocol

To account for indirect cytotoxicity indicated by pH shifts, the pH of the media was measured hourly in the presence of BG particles. All measurements were performed at 37 °C and 5% CO_₂_ using a calibrated pH meter (Mettler Toledo, Switzerland).

### Fluorescence microscopy

After treatment, the wells were rinsed with PBS to remove non-adherent cells and BG particles. For the live/dead assay, the adherent cells were first stained with 100 µL of fluorescein diacetate (FDA, 1.0 µg/mL; Invitrogen, USA) and 100 µL of propidium iodide (PI, 1.0 µg/ml; Roth, Germany). Afterwards, the stained cells were imaged via a BZ-X800 microscope (Keyence, Japan) at 4× magnification [61]. Controls included untreated cells, BG-only, and medium-only samples. Five predefined fields per well (centre and four quadrants) were systematically selected with the microscope’s motorized stage. Acquisition parameters were identical across all groups. Overlapping or clustered cells were segmented using the BZ-X Analyzer’s watershed algorithm (separate area). Non-cellular debris was excluded via circularity and area filters. This semi-automated pipeline ensured consistent quantification across replicates. A representative segmented image is provided in Additional file 1. To assess potential dye interference, BG particles incubated with FDA/PI in the absence of cells were imaged. Minor green-channel autofluorescence was observed at high BG concentrations but excluded by nuclear segmentation. Cell viability (%) was calculated as (live cells/total cells) × 100.

### Flow cytometry

Similarly after 3 h and 72 exposure to BG particles, the wells were gently rinsed with PBS to remove non-adherent cells and particulates. Residual particles were removed by gentle aspiration, and the adherent cells were harvested after treatment with 0.125% trypsin (Gibco, UK), resuspended in 1 mL of complete medium, and transferred to 3-mL tubes. Cells were centrifuged, the supernatant discarded, and resuspended in Ringer’s solution (B. Braun, Germany), which contained Hoechst 33,342 (1.0 µg/mL; Invitrogen, USA), Annexin V-FITC (1 µg/mL; ImmunoTools, Germany), propidium iodide (PI, 50 µg/mL; Roche Diagnostics, Germany), and 1′,3,3,3′,3′-hexamethylindodicarbo-cyanine iodide dye (DilC1, 1 µg/mL; Invitrogen, USA). Controls included unstained, single-stain, and DMSO-treated (positive control) samples. The samples were incubated for 20 min at 4 °C in the dark and analysed via a NovoCyte Advanteon 3-laser flow cytometer (NovCyte Advanteon, Agilent, USA). The cell populations were first gated using R§ forward and side scatter parameters to exclude debris and aggregates, followed by fluorescence channels. Gating strategies were optimized using appropriate controls to identify viable (Hoechst⁺/DilC1⁺/Annexin⁻/PI⁻), early apoptotic (Annexin⁺), and necrotic (PI⁺) subpopulations. Instrument settings and gating parameters were held constant across all replicates to ensure comparability. Cell count normalization was done per 10,000 events/sample. Data were acquired and analysed via NovoExpress software version 1.4.1. [45, 49, 61, 62].

### Statistical analysis

The experiments were conducted in triplicate. The mean value was used as the result and reported as the mean ± standard deviation (SD). The data were analyzed via two-way analysis of variance (2-way ANOVA) with the Bonferroni post hoc correction to assess the combined effects of BG particle size and concentration on osteoblast cells. The level of statistical significance was defined at *p* < 0.05 via GraphPad Prism software version 8.3.0 (GraphPad Software Inc., USA), and Pearson correlation coefficients were calculated to assess covariance between FM and FCM data. The precision of cell viability was defined with the coefficient of variation (CV) for the untreated controls.

## Supplementary Information

Additional file 1.

## Data Availability

The datasets used and/or analysed during the current study are available from the corresponding author upon reasonable request.

## Abbreviations

BG: Bioglass 45S5
FCM: Flow cytometry
FM: Fluorescence microscopy
PI: Propidium iodide
FDA: Fluorescein diacetate
Ax5: Annexin V-FITC
DMSO: Dimethyl sulfoxide
SD: Standard deviation
SAOS-2: Sarcoma osteogenic-2 (human osteoblast-like cell line)
PBS: Phosphate buffered saline
CV: Coefficient of variation
ANOVA: Analysis of variance
