# Supplementary material for: Evaluating cell viability assessment techniques: a comparative study of flow cytometry and fluorescence microscopy in response to bioactive glass exposure
Source: Biomed Eng Online. 2025 Oct 3;24:112. doi: 10.1186/s12938-025-01452-y (PMC12492793; doi:10.1186/s12938-025-01452-y)
Supplement: Supplementary file 1 — Additional file 1. [file 12938_2025_1452_MOESM1_ESM.docx]

# Additional file

# Evaluating cell viability assessment techniques: a comparative study of flow cytometry and fluorescence microscopy in response to bioactive glass exposure

**Bolaji J. Samuel ^1*^, Zhaorui Jin ^2^, Delia S. Brauer ^2^, Georg Matziolis ^1^, Victoria Horbert ^1^**

^1^ University Hospital Jena, Professorship of Orthopaedics at Campus Eisenberg, Friedrich Schiller University Jena, Germany Klosterlausnitzer Strasse 81, 07607 Eisenberg,

^2^ Otto Schott Institute of Materials Research, Friedrich Schiller University, Lessingstraße.12 (AWZ), 07743 Jena, Germany.

Bolaji J. Samuel – **corresponding author**

Email: [bolaji.samuel@med.uni-jena.de](mailto:bolaji.samuel@med.uni-jena.de)

<https://orcid.org/0009-0002-9525-1963>


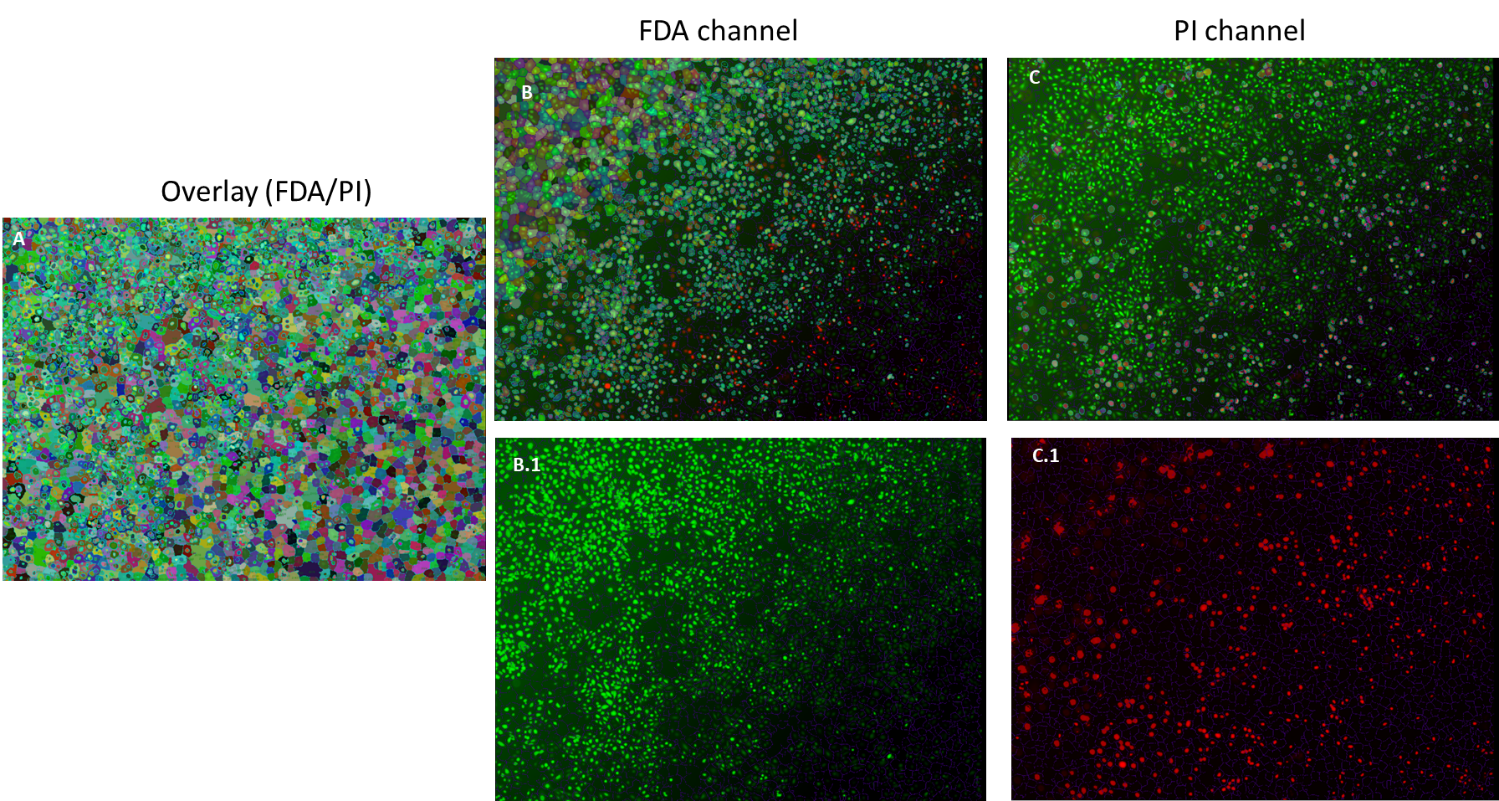


Additional file 1. Representative FM field showing Fluorescein Diacetate (FDA) (green= live cells) and Propidium Iodide Stain (PI) (red = dead cells) fluorescence overlay with segmentation masks applied. The segmentation algorithm used intensity-based thresholding and watershed separation to delineate viable vs. non-viable cells. 4x mag
